# The Immunomodulatory Effect of Vitamin B12 in Pernicious Anemia: A Systematic Review

**DOI:** 10.1155/omcl/8463993

**Published:** 2025-05-26

**Authors:** Tesfaye Engdaw Habtie, Alemu Birara Zemariam, Betelhem Walelgn Dagnaw, Addis Wondmagegn Alamaw, Sefineh Fenta Feleke, Molalign Aligaz Adisu

**Affiliations:** ^1^Department of Nursing, College of Health Sciences, Woldia University, Woldia, Ethiopia; ^2^Department of Pediatrics and Child Health Nursing, College of Health Sciences, Woldia University, Woldia, Ethiopia; ^3^Department of Emergency and Critical Care Nursing, College of Health Sciences, Woldia University, Woldia, Ethiopia; ^4^Department of Public Health, College of Health Sciences, Woldia University, Woldia, Ethiopia

**Keywords:** cyanocobalamin, immunomodulation, NK, pernicious anemia, review

## Abstract

**Objectives:** The aim of this review is to draw attention to key findings from various published studies concerning the effect of methylcobalamin/cyanocobalamin on the immune response of patients diagnosed with pernicious anemia (PA).

**Methods:** This systematic review followed the Preferred Reporting Items for Systematic Reviews and Meta-Analyses (PRISMA) guidelines to ensure the accuracy and reliability of the included randomized controlled trials (RCTs) evaluating the impact of vitamin B12, in either natural or synthetic form, on immune function in patients with PA. The protocol was registered with PROSPERO (CRD42024518621).

**Results:** Methylcobalamin/Cyanocobalamin administration in PA patients significantly increased CD3, CD8+, and CD19 cell levels, restoring them toward normal. Natural Killer (NK) cell activity improved, while the CD4/CD8 ratio decreased. These findings indicate a potential enhancement of immune function in PA patients.

**Conclusion:** Significant restoration of CD3, CD8+, and CD19 cell counts was observed in PA patients after vitamin B12 administration, whether in its natural (methylcobalamin) or synthetic (cyanocobalamin) form. Additionally, NK cell activity was improved, and the CD4/CD8 ratio decreased. These findings suggest that methylcobalamin/cyanocobalamin has the potential to significantly enhance immunity in patients with PA. Therefore, we recommend conducting well-designed, large-scale Phase II and Phase III clinical trials with standardized methodologies to validate these findings and provide more robust evidence on the immunomodulatory effect of vitamin B12 in PA patients.

## 1. Introduction

Vitamin B12 which also called cobalamin, is the largest molecular weight and the most complex micronutrient that have physiological effects on various biological responses. It is widely essential for protein and lipid synthesis, cellular DNA replication, and cell division [1]. It is only synthesized by a small number of microorganisms, however because of the bioaccumulation process, humans can get this vitamin through food [2]. Currently, it has invaluable role in the treatment of pernicious anemia (PA)[3].

PA, an intriguing condition first described by Thomas Addisonin 1849 [4], is a form of megaloblastic anemia that stems from a deficiency of vitamin B12 a vital nutrient for red blood cell production and nerve function [5]. The primary etiology of PA is an autoimmune reaction that targets the gastric parietal cells, leading to a critical shortage of intrinsic factor (IF), a protein essential for vitamin B12 absorption in the ileum. Without this key protein, the body cannot effectively absorb vitamin B12, leading to a deficiency. This can manifest in a range of symptoms, from fatigue and weakness to neurological disturbances and gastrointestinal issues, painting a complex clinical picture [6, 7]. Although rare, PA can be inherited, and it may also be linked to other autoimmune diseases like Addison's disease, Graves' disease, myasthenia gravis, and type 1 diabetes [8].

Vitamin B12, particularly in its active forms, such as methylcobalamin and hydroxocobalamin, has been studied for its potential anti-inflammatory effects in conditions like rheumatoid arthritis and its role in anticancer treatments. It supports DNA synthesis and repair, modulates oxidative stress, and enhances the efficacy of certain chemotherapeutic agents [1].

Recent research has increasingly focused on understanding how individual vitamins, particularly vitamin B12, influence immune function [9]. Despite its essential role in DNA synthesis and neurological function, the immunological mechanisms of vitamin B12 remain unclear. While some evidence suggests its involvement in modulating immune responses, particularly in T cell (TC) regulation and inflammation, further research is needed to establish its definitive role in human immunity [10]. A few studies [10–14] have reported alterations in immunological components in patients with PA following vitamin B12 supplementation. However, some researchers question its impact on human immunity [15], and most previous studies were either poorly designed, animal-based trials or individual case reports [10].

To the best of our knowledge, no systematic review has comprehensively evaluated the effect of vitamin B12 on immune function in PA patients. While several preclinical and clinical randomized controlled trials (RCTs) have explored this relationship, their findings are inconsistent, and individual studies may lack sufficient power to draw definitive conclusions. A systematic review is necessary to synthesize the existing evidence, identify patterns or discrepancies across studies, and highlight areas for further research. This review aims to provide the most comprehensive assessment to date, offering valuable insights into the immunological role of vitamin B12 in PA patients.

## 2. Methods

### 2.1. Data Sources and Searches

This study employs a systematic review, a structured approach to gathering and summarizing existing findings from multiple studies to provide comprehensive evidence on the effect of vitamin B12 on immunity. A comprehensive PROSPERO database search confirmed that no existing reviews on this topic were registered. The study protocol was then registered with PROSPERO (CRD42024518621).

A systematic search of the literature was conducted for RCTs examining the effects of vitamin B12 in its natural (methylcobalamin) or synthetic (cyanocobalamin) forms on immunity in patients with PA. Studies published in reputable, English language journals were included, with no time restrictions, to ensure broad literature coverage. Multiple electronic databases, including PubMed, Google Scholar, and Web of Science, were searched.

The entire review process strictly followed the 2020 Preferred Reporting Items for Systematic Reviews and Meta-Analyses (PRISMA) checklist, ensuring a rigorous, transparent, and unbiased synthesis of the evidence. A meticulously designed search strategy was employed, incorporating carefully selected keywords and Medical Subject Headings (MeSH) terms. Boolean operators (“AND” and “OR”) were strategically applied to refine the search query: (“Immunomodulation” OR “Immune augmentation” OR “Immune enhancement” AND “Cyanocobalamin” OR “Cobalamin” OR “Vitamin B12” OR “Methylcobalamin” AND “Pernicious anemia” OR “Megaloblastic anemia”). These terms were used in various combinations as the primary search keywords. Full-text articles were then assessed for eligibility.

### 2.2. Eligibility Criteria

Two reviewers independently conducted the literature search and resolved any discrepancies through discussion. If additional information was required, the authors contacted the corresponding authors. Eligible RCTs were those that examined the effect of vitamin B12 in its natural (methylcobalamin) or synthetic (cyanocobalamin) form on immunity in individuals with PA and reported changes in CD4+ and CD8+ immune cells, the CD4+/CD8+ ratio, or natural killer (NK) immune cells following vitamin B12 administration. Studies were excluded if they: (1) assessed treatments involving mixed vitamins (e.g., vitamin D and B12 combined); (2) were preclinical RCTs; (3) lacked a robust methodological framework (e.g., absence of randomization, blinding, or appropriate controls); (4) did not provide sufficient data for analysis; (5) were published in languages other than English; or (6) did not provide full-text access. The methodological quality of the included studies was assessed by two independent reviewers using the Risk of Bias 2 (RoB 2) tool, which evaluates five key domains—bias arising from the randomization process, bias due to deviations from intended interventions, bias due to missing outcome data, bias in the measurement of the outcome, and bias in the selection of the reported result [16]. The most methodologically sound RCTs involving humans were then included.

### 2.3. Study Selection

To eliminate duplicate studies, all retrieved records were imported into EndNote version 7 for reference management. Two independent reviewers screened the titles and abstracts based on the predefined selection criteria. Any disagreements were resolved through discussion. Subsequently, a full-text review was conducted independently by two reviewers to determine the final eligibility of studies.

### 2.4. Data Extraction

Using a structural data extraction form, two independent reviewers extracted the data. The step was repeated every time variations in data extraction were noticed. If the differences between the data extractors persisted, a third reviewer was brought in.

## 3. Result

**Figure 1:**
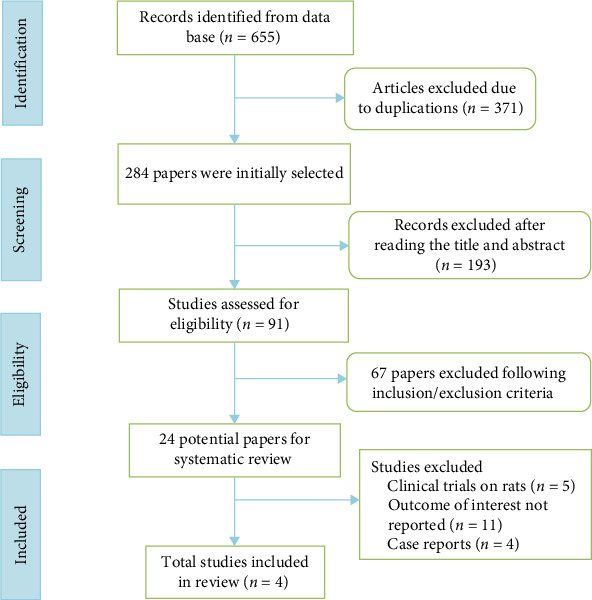
Preferred reporting items for systematic reviews and meta-analyses (PRISMA) flow diagram to illustrate search strategy.

A total of 655 primary studies were identified for possible inclusion through the initial electronic database search. After in-depth review is carried out and duplicates were removed, four eligible RCTs were included in the final analysis (Figure 1).

### 3.1. Characteristics of the Included Studies

All the papers were published between 1984 and 2014 which were divided into three categories: two of the studies used an experimental study group design, one used a prospective research design, and the remaining one study used quasi experimental study design method. The studies' sample sizes varied from 9 to 74. Every study examined the impact of either cyanocobalamin or methylcobalamin on patients' immunity in relation to their PA, either directly or indirectly. The interventions used in the studies all represent a particular micronutrient with its alternative name; vitamin B12, or cyanocobalamin or methyl-B12 (Table 1).

An article by Kätkä [11] examined immunological functions in patients with PA both before and after receiving vitamin B12 treatment. The study participants in this paper were divided into four groups. The first group (5 patients) used Roswell Park Memorial Institute (RPMI 1640) medium and were monitored for 120 days after cyanocobalamin administration. In the second group of six patients, medium RPMI 1640 enhanced with 15% of pooled AB serum was used. In a third group, the same patients were monitored with either RPMI 1640 or special TC 199 medium lacking thymine and folic acid; in a fourth group of eight patients, phytohemagglutinin (PHA) responses were monitored. Throughout the course of their cyanocobalamin treatment, these individuals were monitored for 150 days. The mean age of the treatment group was 65.4 years, and the control group was 44.3 years.

A study conducted by Erkurt et al. [10] aimed to evaluate the role of vitamin B12 in patients with PA and included 30 confirmed patients with a mean age of 55 years (range: 17–75). The diagnosis was established based on medical history, macro-ovalocytosis in peripheral blood, megaloblastic changes in bone marrow, and low serum vitamin B12 levels. In this study, patients received 1000 µg/day of intramuscular cyanocobalamin until a reticulocyte crisis occurred and serum vitamin B12 levels normalized. These values were then compared at the peak reticulocyte count. Tamura's et al. [21] study included 11 cases diagnosed based on medical history, macro-ovalocytosis in peripheral blood, megaloblastic changes in bone marrow, and low serum vitamin B12 levels, along with 13 controls. The mean ages of cases and controls were 65 years (range: 36–83) and 72 years (range: 26–92), respectively. Both groups received 500 µg/day of intramuscular methylcobalamin every other day for 2 weeks. The study assessed leukocyte and lymphocyte counts, absolute numbers and ratios of T and B lymphocyte subgroups, the CD4/CD8 ratio, and NK cell activity before treatment. These values were then compared between cases and controls.

Watanabe et al. [13] followed a different format. The study participants were grouped into three categories: a pretreatment group of 23 confirmed PA patients before treatment started, with a mean age of 66.4 years (range: 36–81); a posttreatment group of 23 patients treated with vitamin B12, with a mean age of 70.3 years (range: 52–94), who had complete hematological remission; and a control group of 28 healthy individuals, with a mean age of 42.3 years (range: 22–65). The study measured the percentages of CD4 and CD8 cells, the CD4/CD8 ratio, Th1/Th2 cells, and Treg cells using fluorescein isothiocyanate (FITC)-labeled anti-CD3, phycoerythrin (PE)-labeled anti-CD4, and CD8-PerCP. Whole heparinized blood was incubated with 25 ng/ml phorbol 12-myristate 13-acetate, 1 μg/ml ionomycin, and 10 μg/ml brefeldin at 37°C with 7% CO_2_ for 4 h. For surface antigen staining, PE-Cy5 antihuman CD4 monoclonal antibody and FITC antihuman CD25 were used. The dose, frequency, and method of administration were not clearly stated in this study.

### 3.2. Outcomes in Relation to the Research Questions

The original question mentioned in this review provides the structure for the presentation of the results. In each instance, both the cases and the control group corresponded to those who get vitamin B12 in either natural or synthetic form.

In a trial conducted by Kätkä [11] to evaluate immune function in PA patients before and during treatment with vitamin B12, peripheral blood lymphocytes were isolated and cultured in vitro under varying conditions to assess immune response. Key parameters included E-rosette forming cells (E-RFC), surface immunoglobulin-positive (S-Ig) lymphocytes, PHA-induced lymphocyte proliferation, and the tuberculin skin test. The lymphocyte cultures were maintained using either RPMI 1640 medium or a thymidine- and folate-free TC 199 medium. The study found that the PHA response was consistently greater in TC 199 medium than in RPMI 1640, particularly in patients who had reached complete remission after 120–180 days of treatment (*p*  < 0.01). Importantly, RPMI 1640 was used solely as a culture medium and was not administered to patients. E-RFC in 9 patients and S-Ig lymphocytes in 5 patients remained normal during therapy. Furthermore, the tuberculin skin test converted from negative to positive in 5 of 7 patients following therapy.

A paper by Erkut et al. [10] reported the status of immunity of patients before and after treatment with cyanocobalamin. After cyanocobalamin treatment, the mean leukocyte count increased from 4432.3 ± 2131.7 to 5416.1 ± 1697.5/mm^3^, and this increase was statistically significant (*p*=0.009). The mean percentage of CD8 cells increased from 26.69 ± 10.23 to 29.42 ± 10.9, with a significant *p*-value of 0.002. The increased CD4/CD8 ratio before treatment decreased from 2.00 ± 1.05 to 1.8 ± 0.83, and the activity of NK cells was restored. The level of C3 increased from 0.75 ± 0.21 to 0.92 ± 0.27, with a *p*-value of 0.001. The levels of immunoglobulins were also elevated: IgG from 10.07 ± 2.77 to 11.28 ± 3.44 (*p*=0.04), IgA from 2.41 ± 1.68 to 3.81 ± 2.67 (*p*=0.01), and IgM from 0.93 ± 0.45 to 1.16 ± 0.57 (*p*=0.03). This implies that cyanocobalamin had immunomodulatory effects in PA patients.

The studies by Tamura et al. [21] and Watanabe et al. [13] consisted of cases and controls. Tamura's [21] found that methyl-B12 treatment considerably enhanced the leucocyte and lymphocyte counts of patients, even though they were lower than in the control groups (*p*  < 0.05). An increase in absolute number and percentage of CD8^+^ cells after methyl-B12 treatment was noted in patients (*p*  < 0.01) and (*p*  < 0.05) respectively but not in control subjects. In patients, the decreased level of NK cell activity was restored by methyl-B12 administration (*p*  < 0.01).When NK cell activity was compared to that seen after 2 weeks of methyl-B12 treatment, patients who had methyl-B12 injections every 3 months to 1–2 years of follow-up showed a greater restoration. While Watanabe et al. [13] demonstrated no statistically significant difference in the mean percentage of CD4+ TCs, among the control groups (41.0%; 95% CI, 37.9%–44.1%), and pre- (43.7%; 95% CI, 36.3 51.1%), and post treatment groups (35.0%; 95% CI, 30.6 39.4%) but there were significant increases in the mean percentage of *T*_reg_ cells in the pre- (6.29%; 95% CI, 5.04%–7.54%) and posttreatment groups (7.77%; 95% CI, 6.34%–9.20%) compared with the control group (4.18%; 95% CI, 3.92%–4.47%; *p*  < 0.05).

## 4. Discussion

Immune cells, such as lymphocytes and macrophages, rely on efficient DNA synthesis and cell division to proliferate and function effectively in combating infections and this is possible when vitamin B12 levels are sufficient. Megaloblastic anemia, neurological disorders of the peripheral nervous system or brain [17], and, in animal models, weakened immunological responses to bacterial and viral infections are the outcomes of vitamin B12 deficiency [18]. Previous studies indicates that vitamin B12 administration improves the immune markers of patients with megaloblastic anemia [18, 19]. Restoring normal levels of vitamin B12 through supplementation with cyanocobalamin can help normalize red blood cell production, improving oxygen transport and overall energy levels [20].This review intends to dig out the role of cyanocobalamin or methylcobalamin in the immunity of PA patients, and in two of the reviewed papers, we noticed different immunomodulatory effects of vitamin B12.

Tamura et al. [21] and Erkurt et al. [10] reported that the diminished NK cell activity in PA patients was restored after administration of methylcobalamin (in Tamura's study) and cyanocobalamin (in Erkurt's study). Both studies observed a significant increase in the mean and absolute number of CD8 (%) cells. In addition, Erkurt's study also reported a significant increase in CD3 (%) and CD19 (%) cells. The mean CD4/CD8 ratio declined after 1 week of methylcobalamin or cyanocobalamin administration in both studies [10, 21]. This implies that vit B_12_ in either natural or synthetic form has enhancement effect of immune cell activity as in CD3 serves as a signal transduction molecule that helps transmit signals from the T-cell receptor (TCR) to the interior of the TC when the TCR binds to its specific antigen. This signaling cascade activates the TC, initiating a series of events that ultimately lead to the TC's response, such as proliferation, cytokine secretion, and direct killing of infected or abnormal cells [22]. CD_8_ cells serves as tissue surveillance, defense and cytotoxic activity by histocompatibility complex class I (MHC-I) molecules which used to detect and kills the infected cells and regulating immune responses through secretion of cytokines, such as interferon-gamma (IFN-*γ*) and tumor necrosis factor-alpha (TNF-*α*).Overall it is essential for maintaining immune homeostasis and protecting the body against a wide range of pathogens and abnormal cells [23, 24]. CD19 cell is a critical molecule which has significant roles in various aspects of B cell biology including development, activation, signaling, and antibody production. Its functions are essential for the generation of effective humoral immune responses, protection against infections, and maintenance of immune homeostasis [25, 26].Kubota et al. [18, 19]. had suportive finding towards the effect of cyanocobalamine on immune modulation in PA patients. A randomized control trial on rats by Funada et al. [27] and Lewicki et al. [28] demonstrated that vitamin B12 has apivotal role in the immunity of anemic rats.

Conversely, Watanabe et al. [13] reported that although there has been a considerable improvement in regulatory TCs, there has been little to no change in CD4, CD8, TH1, TH2, and the TH1/TH2 ratio. Regulatory TC by it self is the most important immune modulator which is used to maintain immune homeostasis. Suppressing unwarranted immunological responses identify and mount responses against foreign antigens while tolerating self-antigens; they also decrease pro-inflammatory immune cell activity, inhibit inflammation, and regulate antitumor immune responses are some of the main roles played by Treg cells [29, 30].The disparity may be related to the method of assigning the participants. In Kätkä's [11] trial with RPMI1640 medium and a specific TC 199 medium lacking thymine and folic acid, it was observed that although the alteration might be linked to the clinical enhancement of the patient, all patients examined had negative tuberculin skin test results prior to cyanocobalamin therapy. However, following the therapies, the test typically yielded positive results. In the trial, there was no observed alteration in immunoglobulin levels, warranting further investigation [11]. Despite the promising findings, this review has some limitations. The included studies varied significantly in sample size and outcome measures, which may impact the comparability and reliability of the results. Some studies did not specify critical details, such as the precise dosage, frequency, and mode of administration of vitamin B12, limiting the ability to draw definitive conclusions. Additionally, small sample sizes in the included studies reduce the statistical power, and the lack of standardized immune assays complicates direct comparisons. Moreover, potential confounders including nutritional status and concurrent medical conditions were not consistently accounted for. Given these limitations, future well-designed, large-scale Phase II and Phase III clinical trials with standardized methodologies are needed to validate these findings and provide more robust evidence on the immunomodulatory effect of vitamin B12 in PA.

## 5. Conclusion and Recommendations

Significant decrease in the number of CD3, CD8+, and CD19 cells were restored in PA patients after vit B_12_ administration with either natural (methylcoblamin) or synthetic (cyanocobalamin) form. The depressed NK cell activity is also improved and the ratio of CD4/CD8 cells becomes reduced. The rise in these biomarkers is a significant indicator of how much vitamin B12 has the potential to enhance immunity. Over all it can protect PA patients from various infectious and noninfectious diseases and it will play an important role in delaying and preventing unexpected deaths. Therefore, we recommend conducting well-designed, large-scale Phase II and Phase III clinical trials with standardized methodologies to validate these findings and provide more robust evidence on the immunomodulatory effect of vitamin B12 in PA.

### 5.1. Limitation

This review has limitations, including small sample sizes and inconsistent reporting of vitamin B12 dosage, frequency, and administration methods, all of which limit comparability and generalizability. The inclusion of studies using varied in vitro assays, such as PHA-induced lymphocyte proliferation tests conducted under different culture conditions (e.g., RPMI 1640 versus TC 199 medium), introduces methodological heterogeneity. These factors collectively highlight the need for well-designed, large-scale clinical trials with standardized methodologies to confirm the immunological effects of vitamin B12 in PA.

### 5.2. Strength

A key strength of this review is the inclusion of RCTs, which enhances the reliability of the findings by minimizing bias, ensuring rigorous control of confounding variables, and providing a higher level of evidence.

## Figures and Tables

**Table 1:** Summary of recent randomized controlled trials (RCTs) investigating the effect of vitamin B12 on immunity.

Reference	Publication year	Country	Method	Intervention	Sample size	Sample size calculated	Method of randomization	Baseline comparability of groups given
Kätkä [11]	1984	Finland	Quasi experimental	Vitamin B12	34	Not stated	Not given	Yes
Tamura et al. [21]	1999	Japan	Experimental control group design	Methyl-B12	24	Not stated	Not given	Yes
Erkurt et al. [10]	2008	Turky	prospective research design	Vitamin B12	30	Not stated	Not given	No
Watanabe et al. [13]	2014	Japan	Experimental control group design	Vitamin B12	74	Not stated	Not given	Yes

## Data Availability

The datasets used and/or analyzed during the current study are available from the corresponding author upon reasonable request.
